# The prognostic value and immune microenvironment association of AR in HER2+ nonmetastatic breast cancer

**DOI:** 10.1038/s41523-023-00527-0

**Published:** 2023-04-21

**Authors:** Danyang Zhou, Mei Li, Mohamed Hussein Yasin, Qianyi Lu, Jia Fu, Kuikui Jiang, Ruoxi Hong, Shusen Wang, Fei Xu

**Affiliations:** 1 Sun Yat-sen University Cancer Center, State Key Laboratory of Oncology in South China, Collaborative Innovation Center for Cancer Medicine, Guangzhou, China; 2 Department of Oncology, Peking University Shenzhen Hospital, Shenzhen, China; 3 Department of Pathology, Sun Yat-sen University Cancer Center, State Key Laboratory of Oncology in South China, Collaborative Innovation Center for Cancer Medicine, Guangzhou, 510000 China

**Keywords:** Breast cancer, Tumour immunology

## Abstract

This study aimed to investigate the prognostic value of AR in HER2+ nonmetastatic breast invasive ductal carcinoma (IDC) and its relationship with the immune microenvironment. HER2+ nonmetastatic breast IDC patients diagnosed by pathology who underwent surgery at Sun Yat-sen University Cancer Center from 2016 to 2017 were included. AR+ and AR− breast IDC samples were matched 1:1 in age, T stage, and N stage for immune infiltration analysis. A total of 554 patients with HER2+ nonmetastatic breast cancer were included in this retrospective study, regardless of HR status. The cut-off value for AR was set at 10%. ER+ (*p* < 0.001) and PR+ (*p* < 0.001) were associated with positive AR expression. Kaplan–Meier survival curve analysis suggested that AR was closely correlated with overall survival (OS) (*p* = 0.001) but not disease-free survival (DFS) (*p* = 0.051). After eliminating the potential impact caused by HR, AR also predicted longer OS (*p* = 0.014) and was an independent predictive factor for OS of HER2+HR− nonmetastatic breast IDC patients, as revealed by multivariate analysis (*p* = 0.036). For AR+ and AR− matched HER2+HR− patients, TILs (*p* = 0.043) and PD-L1 (*p* = 0.027) levels were significantly lower in AR+ patients. The strongest negative correlation was observed between AR and PD-L1 (Pearson’s *r* = −0.299, *p* = 0.001). AR+ status was markedly related to better OS in HER2+HR− nonmetastatic breast cancer patients, while a negative correlation was observed between AR and PD-L1/TILs. We provide new insights into the prognostic value of AR and its association with the immune microenvironment to optimize treatment strategies in HER2+ nonmetastatic breast IDCs.

## Introduction

Breast cancer is the most prevalent cancer that affects women worldwide^1^ and is a heterogeneous disease based on morphological and immunohistochemical subtypes. Among all invasive breast cancers, human epidermal growth factor receptor 2 (HER2)- positive breast cancer accounts for 15–20%^2^ and has distinct biological behaviors and clinical outcomes. HER2 activation occurs through dimerization after binding to ligands, and HER2 signaling activates proliferation, cell survival, metastasis, and adhesion through different pathways^3,4^. In HER2-positive (HER2+) breast cancer, chemotherapy together with anti-HER2 therapy has become the standard of care and is correlated with better outcomes^5–7^.

Androgen receptor (AR) is a steroid hormone nuclear receptor and is widely expressed in breast cancer^8,9^. AR plays inhibitory and stimulatory roles in different types of breast cancer^10,11^. In estrogen receptor (ER)-positive breast cancer, MDV3100 research^12^ has shown that AR signaling is required for both androgen- and estrogen-induced tumor cell growth in vitro and in vivo. In triple negative breast cancer (TNBC), the luminal androgen receptor (LAR) subtype, characterized by the expression of androgen receptor, is associated with better prognosis, less chemotherapy responsiveness, and lower pathologic complete response after neoadjuvant treatment^10^. Notwithstanding the role of AR in several pathways, its impact from a biological and clinical standpoint is still controversial in HER2+ breast cancer. In vivo and in vitro experiments show that inhibition of AR can reduce the growth of HER2+ breast cancer cells and inhibit the phosphorylation of HER2 and the activation of Akt and Erk but does not affect the expression of HER2 protein^13^. Epidemiological evidence suggests that among HER2+ER− patients with nonmetastatic breast cancer, AR+ patients seem to have a worse prognosis^14^. On the other hand, a study including 304 *HER2*-enriched metastatic breast cancer cases indicates that patients with AR+ tumors have a longer progression-free survival (PFS) and a higher 5-year overall survival (OS) rate than those with AR− tumors^15^.

Although breast cancer is considered a cancer with poor immunogenicity^16^, the immune system still plays a key role in the growth and development of breast cancer^17^. The preclinical and clinical data suggest that the response to HER2-targeted therapies^18,19^ and clinical prognosis^20^ can be predicted by immune cell compositions. A study focusing on the interaction between AR and HER2 in breast cancer cell lines indicates that AR signaling regulates direct transcriptional induction of *WNT7B* and *HER3*, resulting in activation of the ligand-dependent Wnt and HER2 signaling pathways^21^. Regarding the relationship among AR level, HER2 and the immune microenvironment, van Rooijen et al. found that in patients with HER2+ metastatic breast cancer receiving trastuzumab treatment, the infiltration of tumor immune cells with low AR expression increased, while the infiltration of tumor immune cells with high AR expression decreased^22^.

Because of the heterogeneity of HER2+ breast cancer and the complex mechanism of HER2 and AR, better understanding of the prognostic value of AR in all HER2+ and HER2+ hormone receptor-negative (HR−) nonmetastatic breast invasive ductal carcinoma (IDC) patients is urgent. In addition, interest in the tumor immune microenvironment is increasing. What is the relationship between AR expression and immune infiltration in HER2+ nonmetastatic breast cancer?

Based on the evidence above, we recruit HER2+ nonmetastatic breast IDC patients diagnosed by pathology who underwent surgery and analyze the prognostic value of AR in HER2+ and HER2+HR− nonmetastatic breast IDC patients. Then, AR+ and AR− breast IDC samples are matched 1:1 according to age, T stage, and N stage to explore their correlation with the immune microenvironment to provide new insights into biological behavior and reasonable treatment strategies.

## Results

### Clinicopathological characteristics and survival of HER2+ patients

**Fig. 1:**
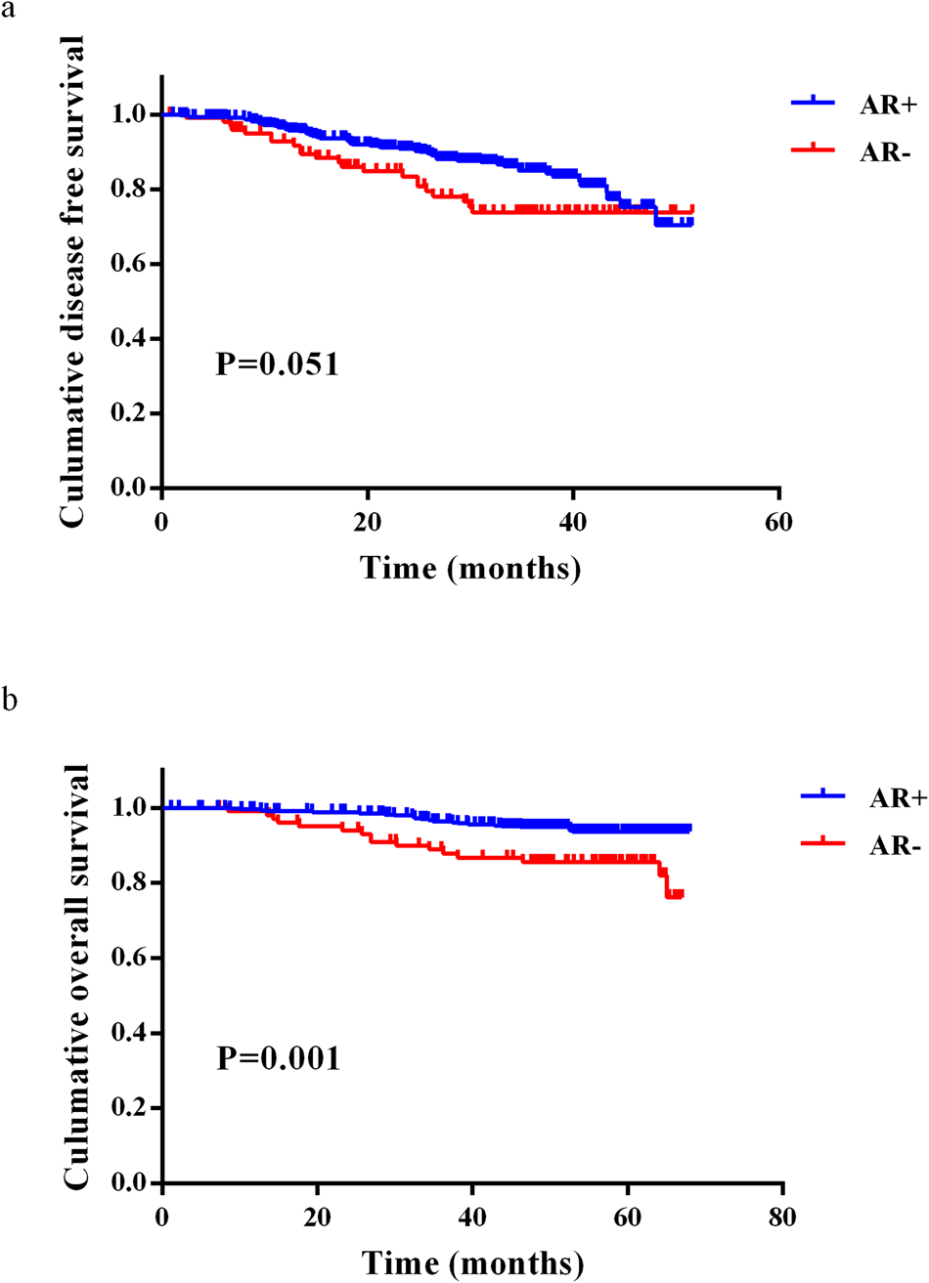
Survival in patients with HER2+ nonmetastatic IDC according to AR expression. Kaplan–Meier curve for DFS (**a**) and OS (**b**) of breast cancer patients stratified by AR status.

**Fig. 2:**
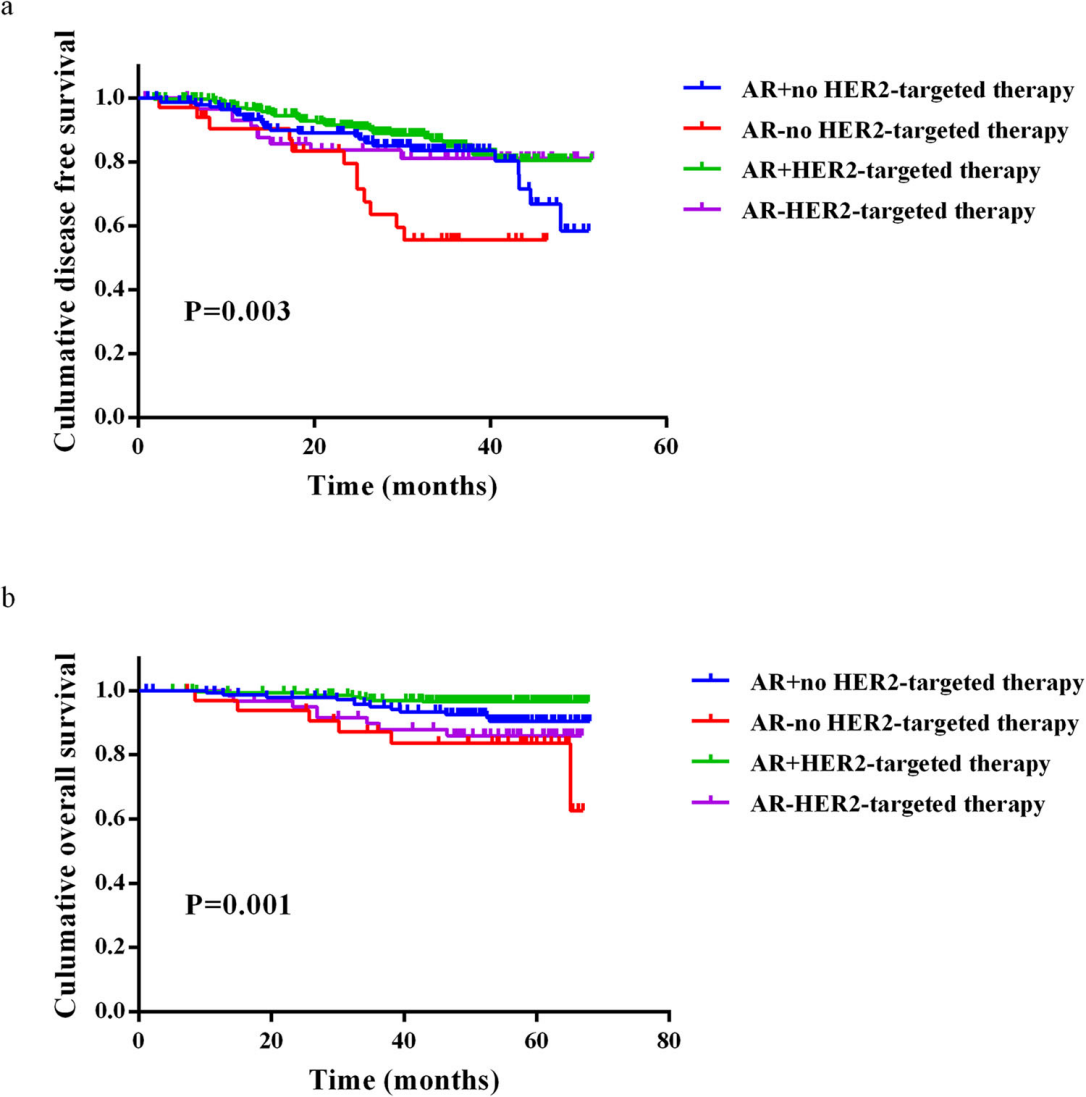
Survival in patients with HER2+ nonmetastatic IDC according to AR expression and HER2-targeted therapy. Kaplan–Meier curve for DFS (**a**) and OS (**b**) of breast cancer patients stratified by AR status and HER2-targeted therapy.

A total of 554 female breast IDC patients were included from 4859 breast cancer patients who visited Sun Yat-sen University Cancer Center between June 2016 and December 2017. The median follow-up after the diagnosis of primary breast cancer was 53.3 months. The median level of AR per tumor was 60.0% (interquartile range (IQR): 20.0–80.0). The cut-off value for AR was set at 10% according to the previous literature and clinical trial^23–28^, 81.6% of patients were AR-positive and 18.4% were AR-negative. Positive expression of ER (*p* < 0.001) and progesterone receptor (PR) (*p* < 0.001) was associated with the high expression of AR using chi-square test. There was also a trend that breast IDC patients in the AR-negative group were older (the cut-off value was the median value of 50 years) (*p* = 0.030) (chi-square test). The clinicopathological data of the included patients are summarized in Table 1. The treatment strategy of patients was determined by the physician based on the patient’s own wishes and their economic situation, especially medical insurance. A total of 473 patients underwent mastectomy, and the rest underwent lumpectomy. For adjuvant/neoadjuvant therapy, doxorubicin/cyclophosphamide followed by paclitaxel were prescribed for the 377 included patients. Only two patients received trastuzumab plus pertuzumab, and the remaining 324 were prescribed trastuzumab monotherapy. There was no significant difference in HER2-targeted therapy, adjuvant/neoadjuvant therapy, or radiotherapy between the AR+ and AR− cohorts. The median disease-free survival (DFS) and OS of these patients were 48.00 and 66.00 months, respectively. Kaplan–Meier survival curve analysis suggested that AR was closely correlated with OS (*p* = 0.001) but not DFS (*p* = 0.051) (Fig. 1), regardless of whether these patients received HER2-targeted treatment (Fig. 2). However, AR was not an independent prognostic factor for DFS and OS, as revealed by multivariate analysis (Supplementary Table 1).

**Table 1:** Clinicopathological characteristics of 554 HER2+ breast IDC patients by chi-square test.

Factor	AR+ (*n* = 452)	AR− (*n* = 102)	*p*
Age			0.030
<50	231	40	
≥50	221	62	
TNM stage			0.259
I–II	312	65	
III	129	35	
Grade			0.597
I–II	159	39	
III	285	62	
ER			<0.001
<1%	204	66	
≥1%	248	36	
PR			<0.001
<1%	242	80	
≥1%	210	22	
Ki67			0.125
<20%	55	7	
≥20%	397	95	
Adjuvant/neoadjuvant therapy			0.406
Yes	391	85	
No	61	17	
HER2-targeted therapy			0.924
Yes	266	60	
No	143	33	
Radiotherapy			0.380
Yes	128	27	
No	286	75

### Clinicopathological characteristics and survival for HER2+HR− patients

**Fig. 3:**
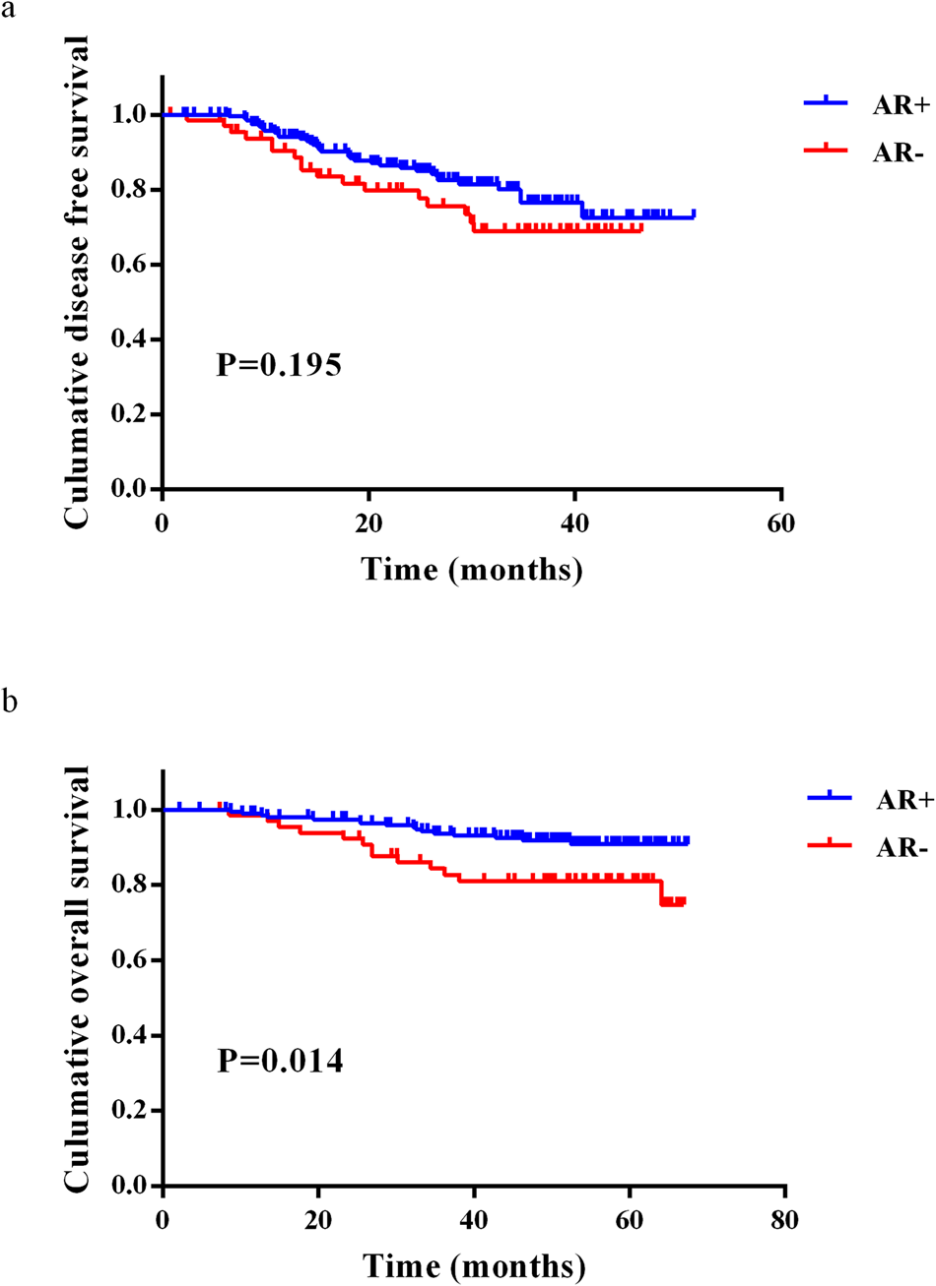
Survival in patients with HER2+HR− nonmetastatic IDC according to AR expression (10%). Kaplan–Meier curve for DFS (**a**) and OS (**b**) of HER2+HR− breast cancer patients stratified by AR status (10%).

**Fig. 4:**
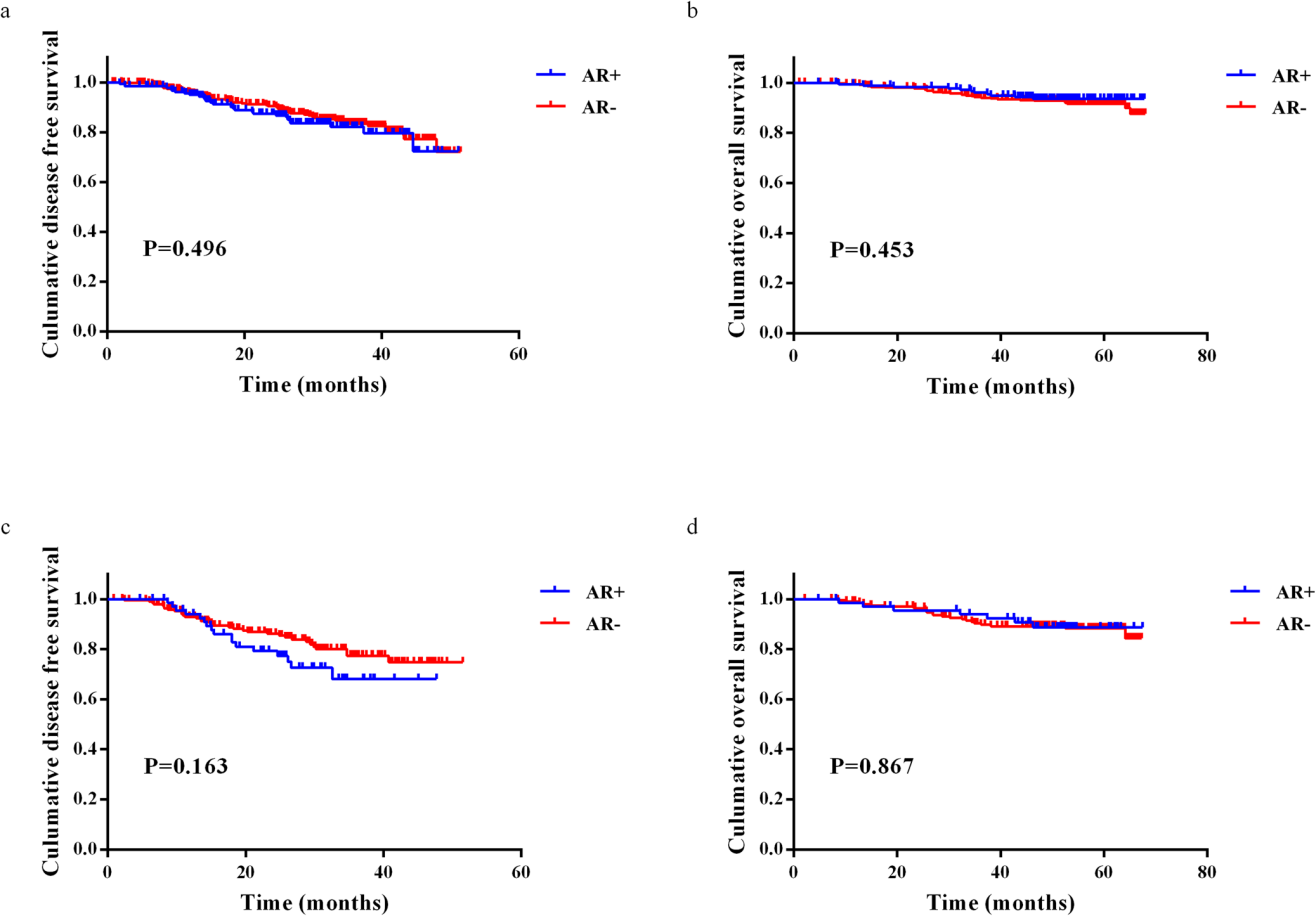
Survival in patients with HER2+ nonmetastatic IDC according to AR expression (78%). **a** Kaplan–Meier curve for DFS of HER2+ breast cancer patients. **b** Kaplan–Meier curve for OS of HER2+ breast cancer patients. **c** Kaplan–Meier curve for DFS of HER2+HR− breast cancer patients. **d** Kaplan–Meier curve for OS of HER2+HR− breast cancer patients.

To eliminate the potential impact caused by HR, we further explored the distribution of AR in clinicopathological values and clinical outcomes in HER2+HR− breast IDC patients. A total of 266 patients of all HER2+ breast IDC patients were HR-negative. A total of 75.2% (200/266) of patients were AR-positive, and 24.8% (66/266) of patients were AR-negative. The percentage of AR− patients among HER2+HR− patients (24.8%) was higher than that among all HER2+ patients (18.4%). The median AR expression was 42.5% (IQR: 8.8–80.0). There was no significant difference in clinicopathological factors or treatment strategies between the AR+ and AR− groups, as shown in Table 2. Similarly, HER2+HR− breast IDC patients who were AR+ had longer OS according to Kaplan–Meier survival curve analysis (*p* = 0.014) (Fig. 3). AR was also an independent prognostic factor for OS in these patients, as revealed by multivariate analysis (*p* = 0.036) (Table 3). Since the optimal cut-off value for AR status is still controversial, we subsequently analyzed the value of 78%, recommended by Ricciardelli et al.^23^, as the AR cut-off values. However, this cut-off value of AR did not predict the survival of all HER2+ and HER2+HR− breast cancer patients (Fig. 4 and Tables 4 and 5).

**Table 2:** Clinicopathological characteristics of 266 HER2+HR− breast IDC patients by chi-square test.

Factor	AR+ (*n* = 200)	AR− (*n* = 66)	*p*
Age			0.555
<50	84	25	
≥50	116	41	
TNM stage			0.821
I–II	129	42	
III	66	23	
Grade			0.411
I–II	46	19	
III	148	47	
Ki67			0.334
<20%	3	3	
≥20%	197	63	
Adjuvant/neoadjuvant therapy			0.057
Yes	181	54	
No	19	12	
HER2-targeted therapy			0.073
Yes	140	39	
No	40	20	
Radiotherapy			0.640
Yes	59	19	
No	126	47

**Table 3:** Cox proportional hazard regression analysis of DFS (A) and OS (B) in HER2+HR− breast IDC patients with AR cut-off of 10%.

Faction	Univariable		Multivariable	
	HR (95% CI)	*p*	HR (95% CI)	*p*
A				
Age (<50 vs. ≥50)	0.634 (0.365–1.100)	0.105		
TNM stage (I–II vs. III)	4.125 (2.313–7.358)	<0.001	4.312 (2.180–8.529)	<0.001
Grade (I–II vs. III)	0.838 (0.458–1.535)	0.568		
AR (<10% vs. ≥10%)	0.681 (0.380–1.223)	0.198		
Ki67 (<20% vs. ≥20%)	0.327 (0.102–1.053)	0.061		
Adjuvant/neoadjuvant therapy (no vs. yes)	1.181 (0.425–3.280)	0.750		
HER2-targeted therapy (no vs. yes)	0.639 (0.348–1.174)	0.149		
Radiotherapy (no vs. yes)	1.896 (1.076–3.341)	0.027	0.907 (0.471–1.747)	0.771

B				
Age (<50 vs. ≥50)	1.858 (0.823–4.196)	0.136		
TNM stage (I–II vs. III)	6.106 (2.699–13.817)	<0.001	10.211 (3.484–29.929)	<0.001
Grade (I–II vs. III)	1.198 (0.486–2.956)	0.695		
AR (<10% vs. ≥10%)	0.410 (0.196–0.856)	0.018	0.408 (0.176–0.944)	0.036
Ki67 (<20% vs. ≥20%)	0.709 (0.096–5.215)	0.736		
Adjuvant/neoadjuvant therapy (no vs. yes)	0.567 (0.216–1.487)	0.249		
HER2-targeted therapy (no vs. yes)	0.413 (0.183–0.930)	0.033	0.504 (0.216–1.176)	0.113
Radiotherapy (no vs. yes)	1.628 (0.747–3.548)	0.220

**Table 4:** Cox proportional hazard regression analysis of DFS (A) and OS (B) in HER2+ breast IDC patients with AR cut-off of 78%.

Faction	Univariable		Multivariable	
	HR (95% CI)	*p*	HR (95% CI)	*p*
A				
Age (<50 vs. ≥50)	0.913 (0.591–1.411)	0.682		
TNM stage (I–II vs. III)	3.406 (2.190–5.297)	<0.001	3.372 (2.148–5.295)	<0.001
Grade (I–II vs. III)	1.086 (0.688–1.714)	0.723		
ER (<1% vs. ≥1%)	0.482 (0.308–0.754)	0.001	0.609 (0.277–1.339)	0.217
PR (<1% vs. ≥1%)	0.501 (0.312–0.804)	0.004	0.712 (0.309–1.642)	0.425
AR (<78% vs. ≥78%)	1.182 (0.750–1.863)	0.470		
Ki67 (<20% vs. ≥20%)	0.719 (0.404–1.279)	0.261		
Adjuvant/neoadjuvant therapy (no vs. yes)	0.967 (0.512–1.827)	0.917		
HER2-targeted therapy (no vs. yes)	0.599 (0.385–0.930)	0.022	0.489 (0.308–0.775)	0.002
Radiotherapy (no vs. yes)	1.537 (0.982–2.407)	0.060		

B				
Age (<50 vs. ≥50)	3.100 (1.520–6.324)	0.002	2.498 (1.154–5.407)	0.020
TNM stage (I–II vs. III)	8.117 (3.977–16.568)	<0.001	12.475 (5.167–30.118)	<0.001
Grade (I–II vs. III)	1.673 (0.814–3.437)	0.161		
ER (<1% vs. ≥1%)	0.348 (0.177–0.683)	0.002	0.704 (0.246–2.015)	0.513
PR (<1% vs. ≥1%)	0.292 (0.135–0.635)	0.002	0.383 (0.109–1.346)	0.134
AR (<78% vs. ≥78%)	0.767 (0.383–1.537)	0.455		
Ki67 (<20% vs. ≥20%)	2.746 (0.661–11.402)	0.164		
Adjuvant/neoadjuvant therapy (no vs. yes)	0.785 (0.348–1.771)	0.559		
HER2-targeted therapy (no vs. yes)	0.510 (0.263–0.991)	0.047	0.413 (0.204–0.837)	0.014
Radiotherapy (no vs. yes)	1.698 (0.884–3.262)	0.112

**Table 5:** Cox proportional hazard regression analysis of DFS (A) and OS (B) in HER2+HR− breast IDC patients with AR cut-off of 78%.

Faction	Univariable		Multivariable	
	HR (95% CI)	*p*	HR (95% CI)	*p*
A				
Age (<50 vs. ≥50)	0.634 (0.365–1.100)	0.105		
TNM stage (I–II vs. III)	4.125 (2.313–7.358)	<0.001	4.312 (2.180–8.529)	<0.001
Grade (I–II vs. III)	0.838 (0.458–1.535)	0.568		
AR (<78% vs. ≥78%)	1.512 (0.842–2.714)	0.166		
Ki67 (<20% vs. ≥20%)	0.327 (0.102–1.053)	0.061		
Adjuvant/neoadjuvant therapy (no vs. yes)	1.181 (0.425–3.280)	0.750		
HER2-targeted therapy (no vs. yes)	0.639 (0.348–1.174)	0.149		
Radiotherapy (no vs. yes)	1.896 (1.076–3.341)	0.027	0.907 (0.471–1.747)	0.771

B				
Age (<50 vs. ≥50)	1.858 (0.823–4.196)	0.136		
TNM stage (I–II vs. III)	6.106 (2.699–13.817)	<0.001	10.402 (3.551–30.475)	<0.001
Grade (I–II vs. III)	1.198 (0.486–2.956)	0.695		
AR (<78% vs. ≥78%)	0.929 (0.396–2.182)	0.867		
Ki67 (<20% vs. ≥20%)	0.709 (0.096–5.215)	0.736		
Adjuvant/neoadjuvant therapy (no vs. yes)	0.567 (0.216–1.487)	0.249		
HER2-targeted therapy (no vs. yes)	0.413 (0.183–0.930)	0.033	0.398 (0.177–0.896)	0.026
Radiotherapy (no vs. yes)	1.628 (0.747–3.548)	0.220		

### Correlation between AR and PD-L1, TILs and Ki-67

**Fig. 5:**
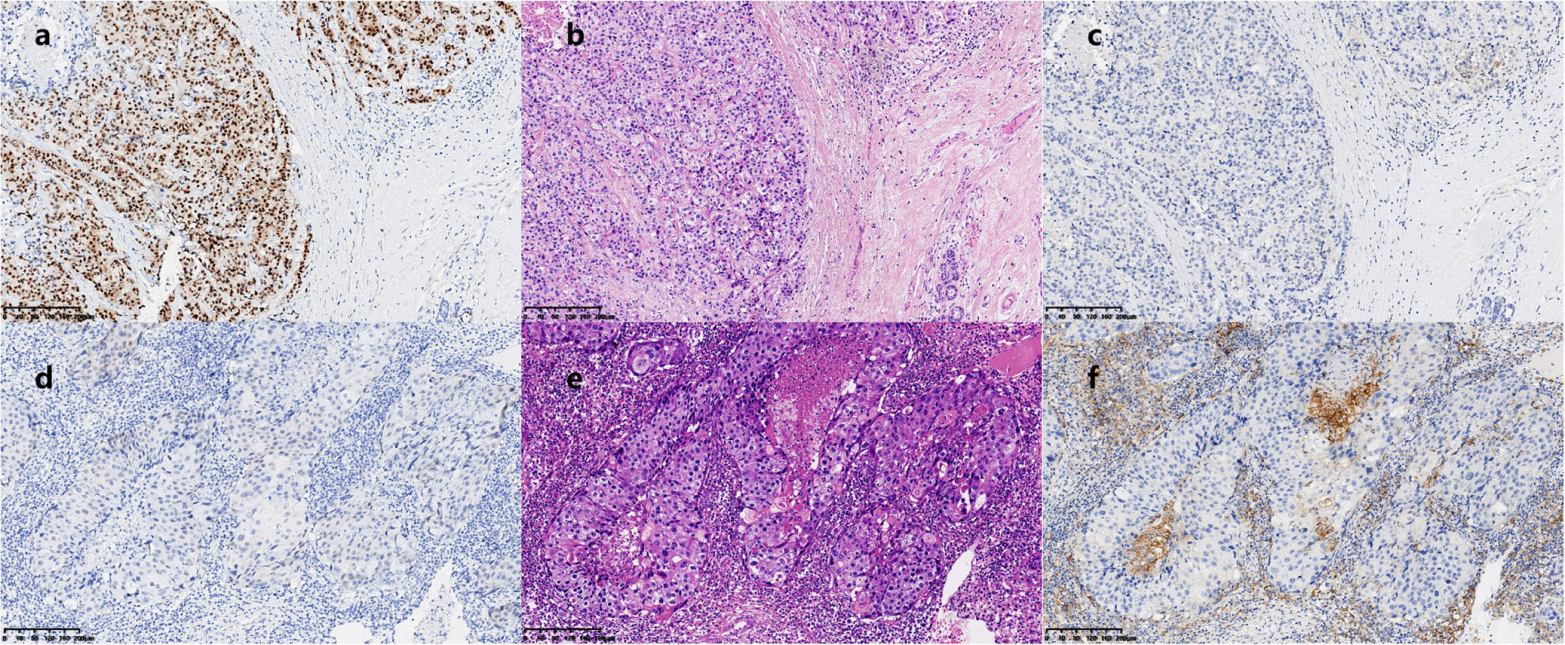
Representative images of TILs and PD-L1 staining in the AR+ and AR− cohorts (×100 magnification). In the AR+ cohort (**a**), the percentages of TILs (**b**) and the expression of PD-L1 (**c**) were low, and in the AR− cohort (**d**), the percentage of TILs (**e**) and the expression of PD-L1 (**f**) were high.

**Fig. 6:**
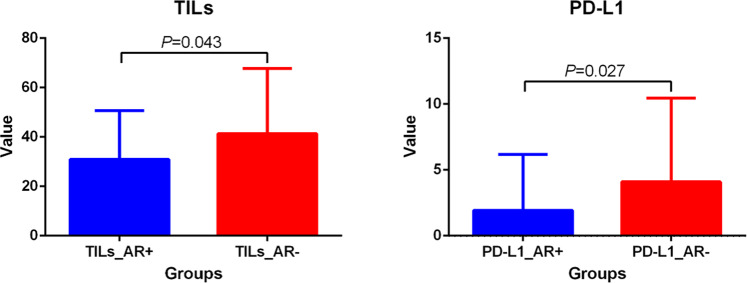
TILs and PD-L1 distribution according to AR expression. Differential distribution of TILs and PD-L1 in matched AR+ and AR− groups using the Wilcoxon signed-rank test. The boxes represent the median value, and the horizontal line represents the standard deviation.

**Fig. 7:**
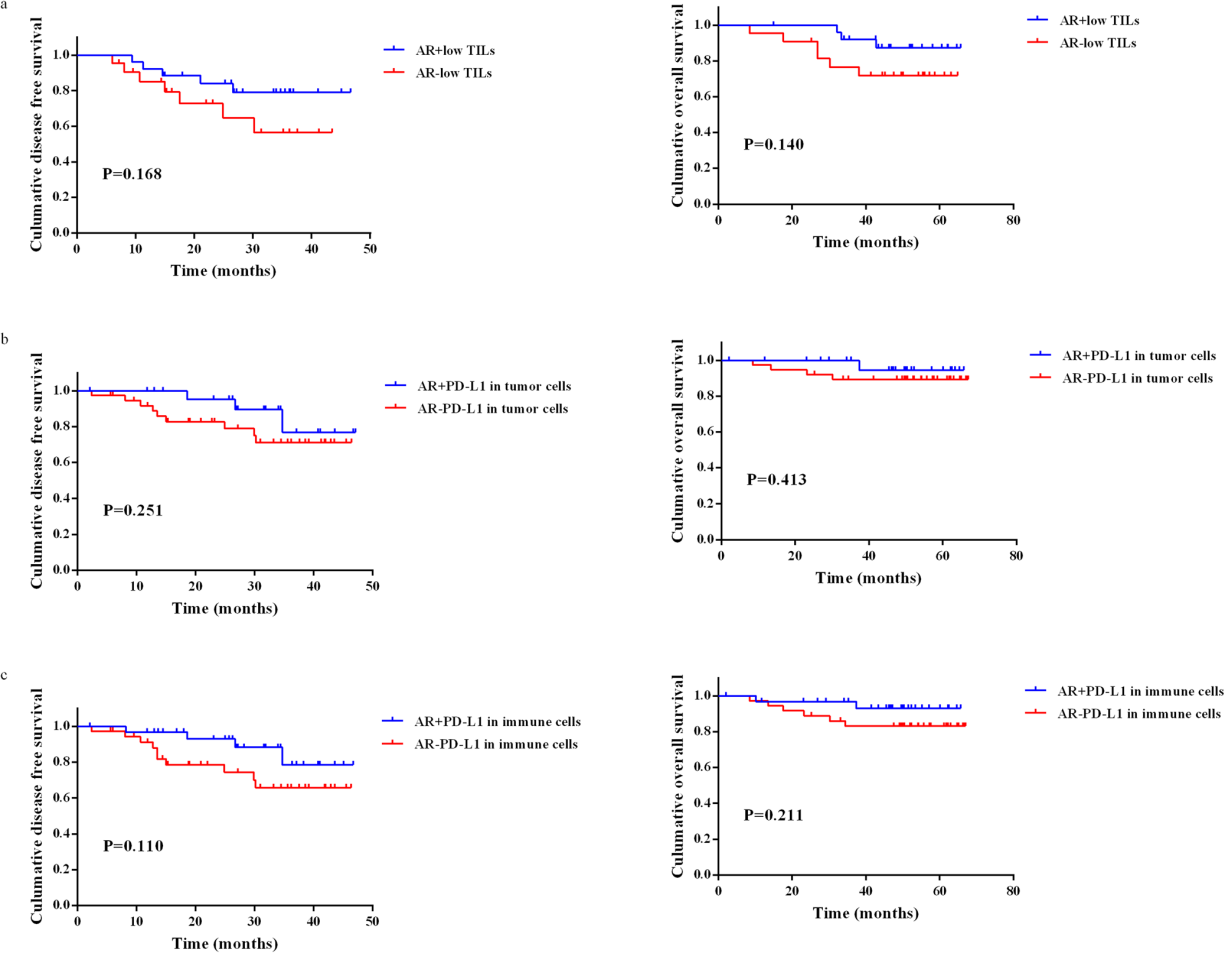
Survival in patients with HER2+HR− nonmetastatic IDC according to AR expression, TILs, and PD-L1. **a** Kaplan–Meier curve for DFS and OS of HER2+HR− breast cancer patients with low TILs stratified by AR status. **b** Kaplan–Meier curve for DFS and OS of HER2+HR− breast cancer patients with PD-L1+ in tumor cells stratified by AR status. **c** Kaplan–Meier curve for DFS and OS of HER2+HR− breast cancer patients with PD-L1+ in immune cells stratified by AR status.

AR+ and AR− samples were matched 1:1 in age, T stage, and N stage in HER2+HR− breast IDC patients. A total of 59 pairs of HER2+HR− patients were selected for immunohistochemistry (IHC), but 3 pairs of patients were excluded in the subsequent analysis due to the absence of pathological specimens. In 56.3% (63 of 112) of the samples, programmed cell death ligand 1 (PD-L1) was expressed by tumor cells. PD-L1 was expressed in 25 tumors in the AR+ group and 38 tumors in the AR− group. PD-L1 was positive in 61.6% (69/112) of immune cells; 33 samples were from AR+ group and 36 were from the AR− group. The median percentages of tumor-infiltrating lymphocytes (TILs) in the AR+ group and AR− group were 30% (IQR: 20–45) and 37.5% (IQR: 20–67.5), respectively. Representative images of TILs and PD-L1 staining in tumor cells in the AR+ and AR− cohorts are shown in Fig. 5. The Wilcoxon signed-rank test suggested that TILs (*p* = 0.043) and PD-L1 (*p* = 0.027) in tumor cells were significantly lower in HER2+HR− breast cancer patients in the AR+ group, as shown in Fig. 6. There was no significant difference in PD-L1 expression in immune cells between the AR+ group and AR− group by the Wilcoxon signed-rank test (*p* = 0.348). The Spearman correlation coefficient indicated that the strongest inverse relationship was found between AR and PD-L1 in tumor cells (Pearson’s *r* = −0.299, *p* = 0.001), followed by AR and TILs (Pearson’s *r* = −0.248, *p* = 0.009), and there was no obvious correlation between PD-L1 in immune cells (*p* = 0.204) or Ki-67 (*p* = 0.793) and AR in HER2+HR− breast IDC patients. In patients with low TILs (the cut-off value was the median value of 30%), patients with high levels of AR in primary tumors were more likely to have a longer DFS (*p* = 0.168) and OS (*p* = 0.140) than those with low levels of AR using Kaplan–Meier survival curve analysis, although there was no significant difference (Fig. 7a). There was also a trend that HER2+HR− breast IDC patients who had positive PD-L1 in both tumor cells and immune cells with high AR expression had a longer DFS and OS than those with low AR expression (Fig. 7b, c).

## Discussion

Androgen receptor is frequently expressed in breast cancer and might be a predictive or prognostic factor and a drug target. However, the prognostic value of AR in HER2+ breast cancer and the relationship between AR and the immune microenvironment are controversial. Our study not only confirmed the prognostic role of AR in all HER2+ nonmetastatic breast IDCs but also confirmed the favorable prognostic value of AR in HER2+HR− nonmetastatic breast cancers. AR was negatively correlated with the immune microenvironment in patients with HER2+HR− nonmetastatic breast cancer. We explored the association between AR and immune infiltration in HER2+ nonmetastatic breast cancer to deepen the current understanding of this disease, increase the stratification of prognosis and hope to facilitate individualized treatment.

Androgen receptor, as a potential biomarker, has recently received more attention. The prognostic relevance of AR may be different in different breast cancer subtypes. It has been reported that the relationship between AR and HER2 seem to be associated with ER status in all types of HER2+ breast cancer, as a significant association between AR and HER2 was observed in ER-negative tumors but not in ER-positive tumors^29^. Ligand-bound AR binds to the estrogen-related element, leading to cell apoptosis in ER-positive/AR-positive cell lines, while AR binds to androgen-related element in the nucleus, leading to proliferation in ER-negative/AR-positive cell lines^9^. In the present study, AR expression predicted a longer OS not only in HER2+ breast cancer but also in HER2+HR− nonmetastatic breast IDC. This indicated that there was no documented association between the good prognosis of AR+ tumors and other favorable prognostic factors, such as ER and PR positivity. Consistent with our results, another cohort^15^, which included 304 HER2+HR− metastatic breast cancer patients, demonstrated that AR positivity was related to favorable survival and predicted the efficacy of first-line trastuzumab treatment in these patients. Moreover, we also found that the survival of AR+ patients was better than that of AR− patients, regardless of whether AR− patients received HER2-targeted treatment. It was further indicated that positive AR was a good prognostic factor of HER2+ breast cancer. Because our results and previous studies demonstrate that AR expression has prognostic value, it is recommended that AR status should be evaluated for patients with *HER2*-enriched breast cancer upon initial diagnosis to obtain comprehensive information. Interestingly, a meta-analysis^30^ revealed that the positive expression of AR was related to DFS and OS in all types of breast cancer patients, while worse clinical outcomes were conferred by AR expression in patients with *HER2*-enriched breast cancer. Gene analysis in this study indicated that in *HER2*-enriched breast cancers, OS with AR mRNA expression was significantly better. In addition, the standardized cut-off for AR prognostication has not been statistically defined. We reviewed the literature and clinical trials^23–28^ and set 10% as the cut-off value for AR, which predicted the prognosis of HER2+ breast cancer patients with statistical significance in the current study. We also tried to use 78% as the cut-off value for AR as recommended by a systematic analysis based on multiple studies^23^. Unfortunately, in the current study, this cut-off value could not predict the clinical outcomes of the included patients.

Since only a fraction of HER2+ breast cancer patients are responsive to the combination of anti-HER2 therapies and immune checkpoint inhibitors, a more comprehensive recognition of the composition of immune infiltration in the tumor microenvironment seems to be helpful in predicting treatment response and optimizing treatment strategy. This study demonstrated that the AR level was inversely related to TILs and PD-L1 in primary HER2+HR− nonmetastatic breast cancer. One study suggested that the level of AR was negatively associated with immune infiltration (M2 tumor-associated macrophages, CD3+ and CD8+ T-cell) in HER2+ metastatic breast cancer^22^, which was consistent with our results in HER2+ nonmetastatic breast cancer. At the same time, it was also found that among patients with high immune infiltration, patients with high levels of AR in primary tumors had longer OS than those with low levels of AR^22^. Another study considering AR and the immune microenvironment was carried out on 107 patients with early and locally advanced TNBC, and it was found that AR expression was also associated with a low immune response, as revealed by cluster analysis of RNA gene expression profiling of primary tumors^31^. AR expression was consistent with an immune-deserted environment based on our findings and previous studies on HER2+ metastatic breast tumors and TNBCs. It was indicated that immunotherapy may have potential implications for tumors with low AR expression and rich immune cells. In addition, a low level of AR was correlated with decreased sensitivity to anti-HER2 therapies, as revealed by a preclinical study focusing on the impact of AR signaling on HER2+ER− breast cancer cells. The most likely cause was that HER2/HER3 signaling activation mediated androgen impairment and stimulating tumor cell growth^21^. Studies on the correlation between AR and PD-L1 have all focused on TNBC. AR+/FOXA1+ tumors exhibited less frequent PD-L1 expression in TNBC^32^. TNBC lacking AR expression was considered quadruple negative breast cancer, where the immune checkpoint inhibitor PD-L1 was significantly upregulated^33^.

It is apparent that androgens and AR interact with each other and affect the biology of HER2+ breast cancer, which deserves more clinical and translational research. Currently, AR is being actively investigated as a therapeutic target of HER2+ breast cancer. A single-arm phase II clinical trial (NCT02091960) exploiting anti-AR in combination with anti-HER2 for AR-positive/HER2-positive/HR-negative MBC is underway. Although this study does not explore immune infiltration, the findings may also provide additional implications for clinical trials.

The limitations of our research are as follows. The optimal cut-off value for AR status remains disputed, varying from >1% to >75%. According to the systematic analysis and previous clinical studies, a threshold of 10% was usually recommended and showed independent prognostic and predictive value. We also tried to use 78% as the cut-off value of AR, but this cut-off did not effectively predict survival in patients with HER2+ breast cancer. Additional limitations of the study include its retrospective nature and the small sample size of AR-negative cases. Considering the length of follow-up and the consistency of treatment strategies, we retrospectively included the data of breast cancer patients who visited our cancer center from 2016 to 2017. Retrospective analysis may have missing or erroneous data entry. Some subgroups analyzed may have insufficient sample sizes to identify significant differences. Therefore, further large-scale prospective studies are needed to confirm our findings. In addition, unveiling the complicated interactions between AR and the HER2 signaling pathway will further help utilize AR as an attractive therapeutic target of *HER2*-enriched nonmetastatic breast cancer.

The positive expression of AR predicted a favorable clinical outcome in HER2+ nonmetastatic breast IDC. In HER2+HR− nonmetastatic breast cancers, AR was negatively correlated with the immune microenvironment. New insights into the association between AR and immune infiltration in HER2+ nonmetastatic breast cancer can be explored to facilitate the stratification of prognosis and provide a reference for individualized treatment.

## Methods

### Patients

Considering the length of follow-up and the uniformity of the treatment strategy, patients with breast cancer at Sun Yat-sen University Cancer Center between June 2016 and December 2017 were retrospectively reviewed. Inclusion criteria were as follows: (1) HER2+ breast IDC diagnosed by pathology, (2) total mastectomy or lumpectomy with a negative margin of surgery, (3) patients without distant metastasis (including distant lymph node metastasis) at initial diagnosis; and (4) sufficient primary tumor pathological tissues for the subsequent immune test. If the patient had a second primary malignancy, the patient was excluded from this study. The AR test was performed on all patients. The following clinicopathologic information was retrieved from medical records: (1) age at diagnosis; (2) TNM stage according to the American Joint Committee on Cancer (AJCC) Cancer Staging Manual 8th Edition^34^; (3) histologic grade of primary tumor; (4) pathological histotype (including ductal and other); (5) ER, PR, HER2, Ki67, and AR status of primary tumor; and (6) treatment strategy (including previous history of surgery, adjuvant/neoadjuvant chemotherapy, HER2-targeted therapy, and radiotherapy). DFS was defined as the interval from surgery for the primary tumor to disease recurrence or metastasis after curative surgery, and OS was defined as the interval from surgery of the primary tumor to death (all causes). All patients were followed up until death or study data cut-off (June 2021). The study was approved by the Ethical Committees of Sun Yat-sen University Cancer Center (No. B2021-266-01). Informed consent was obtained as part of usual clinical practice from subjects involved in the retrospective study, and an informed consent waiver was granted if the patients died or were lost to follow-up.

### Markers’ assessment

The ER, PR, HER2, Ki67, and AR status of the primary tumor were assessed by immunohistochemistry staining and collected from the pathological report. Immunostaining for ER, PR, and Ki67 was evaluated according to the ASCO guidelines^35^, and HER2 was assessed by immunostaining and/or in situ hybridization. ER and PR were considered positive when ≥1% of cells were stained. HER2 was expressed as the recommended 0–3+ by immunostaining evaluation. When the score was 3+, HER2 was considered positive. The tumor was considered HER2-negative and excluded with an IHC score of 0 or 1+, regardless of the in situ hybridization result. Fluorescence in situ hybridization (*HER2* gene amplification detection kit, F01009, China Medical Technologies, Beijing, China. Gene name: GLP *HER2/CSP 17*; probe: *HER2*:17q11.2-q12, *CSP17*:17p11.1-q11.1) was carried out in cases with a score of 2+. A *HER2* gene/chromosome enumeration probe 17 (CEP17) ratio ≥2.0 and an average *HER2* gene copy number ≥4.0 per tumor cell or an average *HER2* gene copy number per cell ≥6.0 were considered HER2-positive results. For Ki67, positive expression revealed by immunohistochemical staining was noted; high expression was defined as a percentage of stained cells ≥20% and low expression was defined as a percentage of stained cells <20%. Approximately AR ≥ 10% of the nuclear stained cells was considered positive. All biomarkers were assessed by immunohistochemistry staining in the Department of Pathology of Sun Yat-sen University Cancer Center. Immunostainings of all five biomarkers were performed using the Ventana Benchmark XT® staining system (Ventana Medical Systems, Tucson, AZ, USA) with the Optiview® DAB Detection Kit (Ventana Medical Systems, Tucson, AZ, USA). ER (clone Sp1, cat. no. 790-4324, Ventana Medical Systems, Tucson, AZ, USA. Dilution: 1:50), PR (clone 1E2, cat. no. 790-2223, Ventana Medical Systems, Tucson, AZ, USA. Dilution: 1:50), HER2 (clone 4B5, cat. no. 790-2991, Ventana Medical Systems, Tucson, AZ, USA. Dilution: 1:250), Ki67 (clone MIB-1, cat. no. IS62630-2, Dako, Santa Clara, California. Dilution: 1:80) and AR (clone EP120, cat. no. ZA-0554, Zsbio, Beijing, China. Dilution: 1:200) antibodies were utilized.

AR+ and AR− patients with HER2+HR− breast IDC were matched 1:1 in age, T stage, and N stage for subsequent analysis. The expression of markers in formalin-fixed paraffin-embedded (FFPE) tissues was quantified, and haematoxylin and eosin (H&E) and immunohistochemistry staining were carried out in one batch per marker to avoid differences in intensity. The expression of PD-L1 was assessed in both tumor cells and immune cells. The percentage of stained tumor cells (tumor cell proportion score, TPS) was regarded as PD-L1 expression in tumor cells, and the percentage of stained immune cells (immune cell proportion score, IPS) was reported as PD-L1 expression in immune cells. PD-L1 IHC testing was performed using the E1L3N antibody (clone E1L3N, cat. no. 13684, Cell Signaling Technology, Danvers, MA, USA; dilution: 1:200) in the biomarker cohort. When ≥1% of cells were stained, PD-L1 was considered positive. According to a standardized method developed by the international TILs working group^36^, TILs were reported for the stromal compartment (=% stromal TILs). The denominator used to determine the % stromal TILs was the area of stromal tissue, not the number of stromal cells. All mononuclear cells (including lymphocytes and plasma cells) were scored, excluding TILs outside of the tumor border; around ductal carcinoma in situ (DCIS) and normal lobules; and in tumor zones with crush artifacts, necrosis, and regressive hyalinization. A full assessment of average TILs in the tumor area by the pathologist was used, not including hotspots. The results are shown as a continuous parameter in as much detail as the pathologist feels comfortable with. The average percentages of PD-L1 and TILs by two observers were used as the final score for every sample. If the difference was greater than 20%, the two observers discussed until reaching an agreement.

### Statistical analysis

Categorical variables are described by percentages, and continuous variables are represented by medians and ranges. The chi-square test’s phi coefficient or Spearman’s correlation coefficient was used to evaluate the relationship among the studied markers. Propensity score matching was used to select matched AR+ and AR− patients. The Wilcoxon signed-rank test was used for the statistical analysis of variation in study markers between paired tissues. The impact of clinicopathologic factors on DFS and OS was calculated by Kaplan–Meier curves. The Cox proportional hazards model was used to evaluate the simultaneous influence of all covariates on DFS and OS. *p* values < 0.05 were considered statistically significant, and all *p* values were two-sided. Statistical analysis was conducted using Statistical Package for the Social Sciences (SPSS), version 25.0.

### Reporting summary

Further information on research design is available in the Nature Research Reporting Summary linked to this article.

## Supplementary information

Reporting Summary

Supplementary Table 1

## Data Availability

The datasets used and/or analyzed during the current study are available from the corresponding author on reasonable request.
