# Supplementary material for: The prognostic value and immune microenvironment association of AR in HER2+ nonmetastatic breast cancer
Source: NPJ Breast Cancer. 2023 Apr 21;9:30. doi: 10.1038/s41523-023-00527-0 (PMC10121570; doi:10.1038/s41523-023-00527-0)
Supplement: Supplementary file 1 — Reporting Summary [file 41523_2023_527_MOESM1_ESM.pdf]

nature portfolio

Shusen Wang Fei Xu

Corresponding author(s): Shusen Wang, Fei Xu

Last updated by author(s): 2023-03-15

Reporting Summary

Nature Portfolio wishes to improve the reproducibility of the work that we publish. This form provides structure for consistency and transparency in reporting. For further information on Nature Portfolio policies, see our Editorial Policies and the Editorial Policy Checklist.

Statistics

For all statistical analyses, confirm that the following items are present in the figure legend, table legend, main text, or Methods section.

n/a	Confirmed	
☐	☒	The exact sample size (n) for each experimental group/condition, given as a discrete number and unit of measurement
☐	☒	A statement on whether measurements were taken from distinct samples or whether the same sample was measured repeatedly
☐	☒	The statistical test(s) used AND whether they are one- or two-sided<br>*Only common tests should be described solely by name; describe more complex techniques in the Methods section.*
☐	☒	A description of all covariates tested
☒	☐	A description of any assumptions or corrections, such as tests of normality and adjustment for multiple comparisons
☐	☒	A full description of the statistical parameters including central tendency (e.g. means) or other basic estimates (e.g. regression coefficient) AND variation (e.g. standard deviation) or associated estimates of uncertainty (e.g. confidence intervals)
☒	☐	For null hypothesis testing, the test statistic (e.g. F, t, r) with confidence intervals, effect sizes, degrees of freedom and P value noted<br>*Give P values as exact values whenever suitable.*
☒	☐	For Bayesian analysis, information on the choice of priors and Markov chain Monte Carlo settings
☒	☐	For hierarchical and complex designs, identification of the appropriate level for tests and full reporting of outcomes
☒	☐	Estimates of effect sizes (e.g. Cohen's d, Pearson's r), indicating how they were calculated

Our web collection on statistics for biologists contains articles on many of the points above.

Software and code

Policy information about availability of computer code

Data collection	No software was used.
Data analysis	No software was used.

For manuscripts utilizing custom algorithms or software that are central to the research but not yet described in published literature, software must be made available to editors and reviewers. We strongly encourage code deposition in a community repository (e.g. GitHub). See the Nature Portfolio guidelines for submitting code & software for further information.

Data

Policy information about availability of data

All manuscripts must include a data availability statement. This statement should provide the following information, where applicable:

- Accession codes, unique identifiers, or web links for publicly available datasets
- A description of any restrictions on data availability
- For clinical datasets or third party data, please ensure that the statement adheres to our policy

The datasets used and/or analyzed during the current study are available from the corresponding author on reasonable request.

Field-specific reporting

Please select the one below that is the best fit for your research. If you are not sure, read the appropriate sections before making your selection.

[x] Life sciences [ ] Behavioural & social sciences [ ] Ecological, evolutionary & environmental sciences

For a reference copy of the document with all sections, see nature.com/documents/nr-reporting-summary-flat.pdf

Life sciences study design

All studies must disclose on these points even when the disclosure is negative.

Sample size	All breast cancer patients who met the inclusion criteria and visited Sun Yat-sen University Cancer Center between June 2016 and December 2017 were included in the study.
Data exclusions	If the patient had a second primary malignancy, the patient was excluded from this study.
Replication	More than 2 authors participated in data collection, verification and analysis. The percentages of PD-L1 and TILs were averaged by two observers and used as the final score for every sample. All attempts at replication were successful.
Randomization	This is not relevant to our study. According to the previous literature and clinical trials, taking 10% as the critical value of AR, the included patients were divided into AR positive and AR negative groups in the present study.
Blinding	The investigators participated in data collection, verification and analysis independently. Two pathologists independently evaluated the expression of PD-L1 and TILs without knowing the patient's clinical data.

Reporting for specific materials, systems and methods

We require information from authors about some types of materials, experimental systems and methods used in many studies. Here, indicate whether each material, system or method listed is relevant to your study. If you are not sure if a list item applies to your research, read the appropriate section before selecting a response.

Materials & experimental systems

n/a	Involved in the study	
[ ]	[x]	Antibodies
[x]	[ ]	Eukaryotic cell lines
[x]	[ ]	Palaeontology and archaeology
[x]	[ ]	Animals and other organisms
[ ]	[x]	Human research participants
[ ]	[x]	Clinical data
[x]	[ ]	Dual use research of concern

Methods

n/a	Involved in the study	
[x]	[ ]	ChIP-seq
[x]	[ ]	Flow cytometry
[x]	[ ]	MRI-based neuroimaging

Antibodies

Antibodies used	ER (790-4324, Ventana Medical Systems, Tucson, AZ, USA)<br>PR (790-2223, Ventana Medical Systems, Tucson, AZ, USA)<br>HER2 (790-2991, 4B5, Ventana Medical Systems, Tucson, AZ, USA)<br>Ki67 (IS62630-2, Dako, Santa Clara, California)<br>AR (ZA-0554, Zsbio, Beijing, China)<br>PD-L1 (E1L3N, Cell Signaling Technology, Danvers, Massachusetts, USA)
Validation	*Describe the validation of each primary antibody for the species and application, noting any validation statements on the manufacturer's website, relevant citations, antibody profiles in online databases, or data provided in the manuscript.*

Human research participants

Policy information about studies involving human research participants

Population characteristics	All included patients were female HER2+ breast invasive ductal carcinoma. The patient's ages ranged from 22 to 87 years. Taking 10% as the cutoff value of AR according to the previous literature and clinical trial, 81.6% of patients were AR positive and 18.4% were AR negative. The treatment strategy of patients was determined by the physician, taking into account the patient's own wishes and their economic situation, especially medical insurance. A total of 473 patients received the mastectomy and the rest underwent the lumpectomy. For adjuvant/neoadjuvant therapy, doxorubicin/cyclophosphamide followed by paclitaxel (AC followed by T) was performed in 377 included patients. Only 2 patients received trastuzumab plus pertuzumab and the remaining 324 received trastuzumab monotherapy.

Recruitment

Considering the length of follow-up and the uniformity of treatment strategy, patients with breast cancer at Sun Yat-sen University Cancer Center between June 2016 and December 2017 were retrospectively reviewed. Inclusion criteria were as follows: (1) HER2+ breast invasive ductal carcinoma (IDC) diagnosed by pathology, (2) having received total mastectomy or lumpectomy and achieved the negative margin of surgery, (3) patients without distant organ (including distant lymph node) metastasis at initial diagnosis, and (4) sufficient primary tumor pathological tissue for a subsequent immune test. If the patient had a second primary malignancy, the patient was excluded from this study.
The retrospective feature may lead to the incompleteness of the clinicopathologic records. The single-center study may result in a smaller sample size. These two points may cause bias.

Ethics oversight

The study was approved by the Ethical Committees of Sun Yat-sen University Cancer Center (No. B2021-266-01)

Note that full information on the approval of the study protocol must also be provided in the manuscript.

Clinical data

Policy information about clinical studies

All manuscripts should comply with the ICMJE guidelines for publication of clinical research and a completed CONSORT checklist must be included with all submissions.

Clinical trial registration

The current study is a retrospective study rather than a clinical trial. Our study was approved by the Ethical Committees of Sun Yat-sen University Cancer Center (No. B2021-266-01).

Study protocol

The inclusion and exclusion criteria of patients, , as well as the examination and evaluation methods of various biomarkers (ER, PR, HER2, Ki-67, AR and PD-L1) are described in detail in the manuscript.

Data collection

In the present study, patients with breast cancer at Sun Yat-sen University Cancer Center between June 2016 and December 2017 were retrospectively reviewed. We collected the clinicopathological data of patients from the medical record system. The disease progression and survival of patients were regularly followed up by the follow-up department. The examination and evaluation of ER, PR, HER2, Ki-67, AR and PD-L1 were completed in the pathology department. The time period for study was from August 2021 to February 2022.

Outcomes

We aimed to investigate the prognostic value of AR and the relationship between AR expression and the immune microenvironment in HER2+ non-metastatic breast invasive ductal carcinoma (IDC). We evaluated the survival of patients with different AR expression groups using DFS and OS. The relationship between AR expression and immune microenvironment was evaluated by PD-L1 and TILs.
