# Supplementary material for: The prognostic value and immune microenvironment association of AR in HER2+ nonmetastatic breast cancer
Source: NPJ Breast Cancer. 2023 Apr 21;9:30. doi: 10.1038/s41523-023-00527-0 (PMC10121570; doi:10.1038/s41523-023-00527-0)
Supplement: Supplementary file 2 — Supplementary Table 1 [file 41523_2023_527_MOESM2_ESM.docx]

Supplementary Table 1. Cox proportional hazard regression analysis of DFS (A) and OS (B) in HER2+ breast IDC patients.

A

| Faction | Univariable | | Multivariable | |
| --- | --- | --- | --- | --- |
|  | HR (95% CI) | P | HR (95% CI) | P |
| Age (< 50 vs >= 50) | 0.913 (0.591-1.411) | 0.682 |  |  |
| TNM stage (I-II vs III) | 3.406 (2.190-5.297) | <0.001 | 3.372 (2.148-5.295) | <0.001 |
| Grade (I-II vs III) | 1.086 (0.688-1.714) | 0.723 |  |  |
| ER (<1% vs >= 1%) | 0.482 (0.308-0.754) | 0.001 | 0.609 (0.277-1.339) | 0.217 |
| PR (<1% vs >= 1%) | 0.501 (0.312-0.804) | 0.004 | 0.712 (0.309-1.642) | 0.425 |
| AR (<10% vs >= 10%) | 0.617 (0.378-1.006) | 0.053 |  |  |
| Ki-67 (<20% vs >= 20%) | 0.719 (0.404-1.279) | 0.261 |  |  |
| Adjuvant/neoadjuvant therapy (no vs yes) | 0.967 (0.512-1.827) | 0.917 |  |  |
| HER-2 targeted therapy (no vs yes) | 0.599 (0.385-0.930) | 0.022 | 0.489 (0.308-0.775) | 0.002 |
| Radiotherapy (no vs yes) | 1.537 (0.982-2.407) | 0.060 |  |  |

B

| Faction | Univariable | | Multivariable | |
| --- | --- | --- | --- | --- |
|  | HR (95% CI) | P | HR (95% CI) | P |
| Age (< 50 vs >=50) | 3.100 (1.520-6.324) | 0.002 | 2.286 (1.043-5.007) | 0.039 |
| TNM stage (I-II vs III) | 8.117 (3.977-16.568) | <0.001 | 11.827 (4.892-28.595) | <0.001 |
| Grade (I-II vs III) | 1.673 (0.814-3.437) | 0.161 |  |  |
| ER (<1% vs >= 1%) | 0.348 (0.177-0.683) | 0.002 | 0.688 (0.239-1.977) | 0.487 |
| PR (<1% vs >= 1%) | 0.292 (0.135-0.635) | 0.002 | 0.456 (0.126-1.653) | 0.232 |
| AR (<10% vs >= 10%) | 0.354 (0.188-0.665) | 0.001 | 0.542 (0.266-1.105) | 0.092 |
| Ki-67 (<20% vs >= 20%) | 2.746 (0.661-11.402) | 0.164 |  |  |
| Adjuvant/neoadjuvant therapy (no vs yes) | 0.785 (0.348-1.771) | 0.559 |  |  |
| HER-2 targeted therapy (no vs yes) | 0.510 (0.263-0.991) | 0.047 | 0.452 (0.221-0.924) | 0.030 |
| Radiotherapy (no vs yes) | 1.698 (0.884-3.262) | 0.112 |  |  |
